# Application of a Non-Isothermal Numerical-Analytical Model to Determine the Kinetics of Austenite Formation in a Silicon Alloyed Steel

**DOI:** 10.3390/ma15041376

**Published:** 2022-02-13

**Authors:** Alexis Iván Gallegos-Pérez, Octavio Vázquez-Gómez, Martín Herrejón-Escutia, Héctor Javier Vergara-Hernández, Sixtos Antonio Arreola-Villa, Pedro Garnica-González, Edgar López-Martínez

**Affiliations:** 1 Tecnológico Nacional de México/I.T. Morelia, Tecnológico 1500, Lomas de Santiaguito, Morelia 58120, Mexico; alexis.gp@morelia.tecnm.mx (A.I.G.-P.); h_escuti@hotmail.com (M.H.-E.); hector.vh@morelia.tecnm.mx (H.J.V.-H.); pedro.gg@morelia.tecnm.mx (P.G.-G.); 2 Consejo Nacional de Ciencia y Tecnología, Insurgentes 1582, Crédito Constructor, Ciudad de Mexico 03940, Mexico; 3 Facultad de Ingeniería Mecánica y Eléctrica, Barranquilla s/n, Guadalupe, Monclova 25280, Mexico; svilla@uadec.edu.mx; 4 Universidad del Istmo, Ciudad Universitaria s/n, Barrio Santa Cruz 4a. Secc., Santo Domingo Tehuantepec 70760, Mexico; lopeze@sandunga.unistmo.edu.mx

**Keywords:** austenite formation, kinetics, non-isothermal model, dilatometry

## Abstract

A non-isothermal transformation model was proposed to determine the austenite formation kinetics in a steel alloyed with 2.6% wt. Si by dilatometric analysis, considering that the nucleation mechanism does not change with the heating rate. From the dilatometric analysis, it was observed that the austenite formation occurs in two stages; critical temperatures, degree and austenite formation rate were determined. The activation energies associated with each of the stages were obtained employing the Kissinger method (226.67 and 198.37 kJ·mol^−1^ for the first and second stage) which was used in concert with the austenite formation rate in the non-isothermal model as a first approximation, with acceptable results in the second stage, but not in the first due to the activation energies magnitude. Then, the activation energies were adjusted by minimizing the minimal squares error between estimated and experimental austenite formation degree, obtaining values of 158.50 kJ·mol^−1^ for the first and 165.50 kJ·mol^−1^ for the second stage. These values are consistent with those reported for the diffusion of carbon in austenite-FCC in silicon steels. With these activation energies it was possible to predict the austenite formation degree with a better level of convergence when implementing the non-isothermal model.

## 1. Introduction

The austenite formation is an important part as a previous stage (austenitization) in the heat treatment of steels to obtain microstructures with specific mechanical properties. Through the solid–solid phase transformations and the effect of the alloying elements, it is possible to obtain a specific distribution of phases and micro-constituents while maintaining a synergy between mechanical properties and microstructure. Synergy is established through the relationship between thermal and kinetic parameters associated with the phase transformation mechanisms.

In this sense, it has been shown that austenite formation occurs mainly by nucleation and growth processes through studies where kinetic parameters associated with transformation mechanisms in low carbon steels have been empirically determined [1,2,3]. However, there are studies on other types of steels such as: medium and high carbon [4,5,6], microalloyed steels [7,8], cast irons [9,10,11] and alloys with certain quantity of alloying elements such as silicon, manganese and chromium [12,13,14].

On this basis, it was shown that the austenitic transformation kinetics is highly sensitive to the content of the alloying elements by promoting or delaying the austenite formation owing to the elements partition that occurs during the reaction as a function of the phases and microconstituents present. In analogy to the silicon effect on austenite decomposition kinetics—delay in the precipitation of cementite, [15]—it has been shown that silicon delays the onset of austenite formation, from a microstructure composed of ferrite and pearlite. In addition, this type of transformation is carried out in two stages, contrary to what happens in plain medium-carbon steels where a similar microstructure is presented [14].

On the other hand, the study related to the formation of austenite has been extended to the use of kinetic models to determine the degree of transformation under isothermal and continuous conditions [1,2,3,5,6,7,8,9,10,11,14,16,17]. The implementation of kinetics models depends mainly on the approach; i.e., analytical kinetic models based on the Johnson–Mehl–Avrami (JMA) model aim to describe the coupled transformation mechanisms (nucleation, growth and impingement) under specific kinetic conditions [16,17], while conventional models try to estimate the transformation degree through the change in dilation strain with respect to the phases present by lattice parameters as a function of temperature and composition [3,6,18]. Likewise, there are models that adjust the transformation degree through numerical-analytical models based on assumptions regarding the type of transformation, using some of the kinetic parameter as an adjustment parameter. This allows for improvement of the estimation of the transformation degree, preserving the analytical foundation regarding the transformation mechanism [5].

The JMA model has been commonly used as the basis of kinetics models, describing the transformation mechanisms through its parameters $k_{0}$, n y Q associated with the nucleation and growth rate, geometry and nucleation rate, as well as effective activation energy, respectively. These parameters are a function of time or temperature depending on the treatment condition, isothermal or non-isothermal. The aim of this work is to estimate the austenite formation kinetics by means of a non-isothermal analytical-numerical model based on the JMA model in an experimental medium-carbon steel alloyed with silicon. The use of this model lies in the application of heat treatments in steels, either for transformation routes design in industrial processes, or for the understanding of the mechanisms associated with phase transitions.

## 2. Austenite Formation Models

### 2.1. Isothermal–JMA Model

Solid–solid phase transformations that occur during austenitizing heat treatment in steels are generally described by semi-empirical diffusive models that were developed under isothermal conditions. The JMA model for phase transformations considers the mechanisms of continuous and random nucleation under isothermal conditions, which are expressed as:

(1) $$X=1-\exp\left(-k^{n}\left(T\right)\cdot t^{n}\right)$$

where X is the transformation degree of the phase in formation at time t and T is the specific temperature, while n is the growth exponent, related with geometry and nucleation rate, and k is the kinetic parameter associated to transformation rate (nucleation and growth) depending on the temperature. According to above-mentioned, the values of k and n allow to deduce the mechanisms that govern the phase transformation, and in turn, quantify the rate with which they are carried out [19]. Conversely, the parameter k can be expressed from its dependence with temperature by Arrhenius-type equation,

(2) $$k\left(T\right)=k_{0}\cdot \exp\left(-\frac{Q}{RT}\right)$$

Therefore, the Equation (1) can be expressed as,

(3) $$X=1-\exp\left(-k_{0}^{n}\cdot \exp\left(-\frac{nQ}{RT}\right)\cdot t^{n}\right)$$

where $k_{0}$ is the pre-exponential coefficient of k, Q is the effective activation energy and *R* is the universal gas constant. The JMA model can be applied for the analysis of isothermal and non-isothermal transformations if X is considered in terms of the extended transformed fraction that fully determines the degree of transformation X(0≤X≤1) [16,17]. Besides that, the transformation rate dX/dt can be expressed as the product of two functions that only depend on the transformation degree X and the temperature T, even for the simplest kinetic condition such as the isokinetic Avrami condition [20]. When considering the isokinetic condition, it is assumed that the transformation mechanism does not change with respect to time and temperature, therefore, the kinetic parameters remain constant.

### 2.2. Non-Isothermal-JMA Model

The non-isothermal JMA model is based on the transformation rate, which is considered as a state function independent of the thermal path and when applying the isokinetic Avrami condition is obtained:

(4) $$\frac{dX}{dt}=f\left(X,T\right)=h\left(X\right)\cdot g\left(T\right)$$

where h(X) is a function that only depend of transformation degree and g(T) is a function that depend of the temperature. By deriving Equation (3) with respect to time, the isokinetic condition is partially fulfilled (Equation (4)) by obtaining an expression that depends on time and temperature:

(5) $$\frac{dX}{dt}=-nk_{0}^{n}t^{n-1}\exp\left(-\frac{nQ}{RT}\right)\exp\left(-k_{0}t^{n}\exp\left(-\frac{nQ}{RT}\right)\right)$$

For Equation (5) to fully comply with the isokinetic condition, the time dependence must be eliminated, solving t from Equation (3) and substituting it in Equation (5), therefore:

(6) $$\frac{dX}{dt}=f\left(X,T\right)=-nk_{0}\left(1-X\right)\exp\left(-\frac{Q}{RT}\right)\ln{\left(1-X\right)}^{\frac{n-1}{n}}$$

Equation (6) represents the transformation rate under the isokinetic Avrami condition, therefore, it is assumed that the transformation rate depends only on the transformation degree and temperature, keeping constant the growth exponent n and effective activation energy Q. It should be noted that f(X,T) is a functional that requires a specific time-temperature-path. However, this expression can be conveniently parameterized when T(t) is limited to a constant heating rate (isochronous condition): $T(t)=T_{i}+\varphi (t)$, where $T_{i}$ is the initial temperature [21]. In this case, f(X,T) can be determined for each heating rate such that,

(7) $$\frac{dX}{dt}=f{\left(X,T(t)\right)}_{\varphi }$$

## 3. Materials and Methods

### 3.1. Material

**Figure 1:**
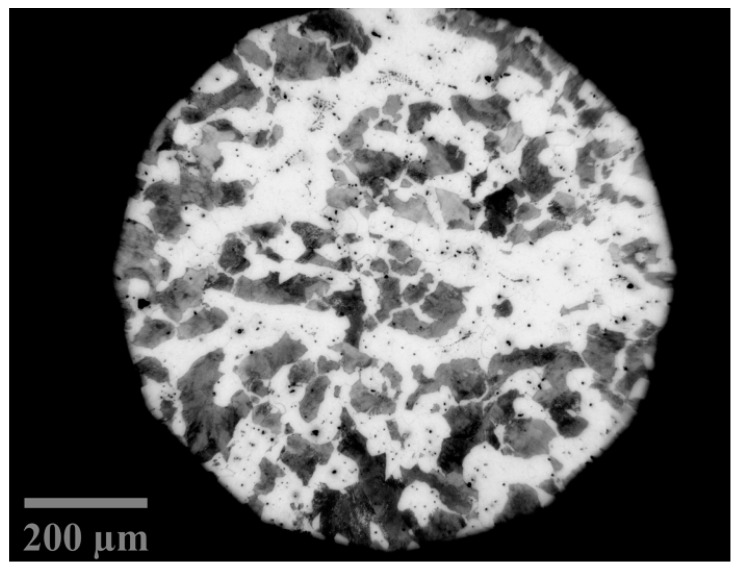
Microstructure of silicon-alloyed steel composed of ferrite (**light zones**) and pearlite (**dark zones**) grains, etched with Nital 3.

Austenite formation kinetics were determined in a silicon-alloyed steel designated 2.6Si, whose chemical composition is indicated in Table 1. Figure 1 shows the initial microstructure of the steel composed of ferrite and pearlite grains with a ferrite volume fraction of 0.507 ± 0.004 and a Vickers microhardness of 237.8 HV0.2/15 according to present microstructure. The steel was prepared by cutting and grinding with SiC sandpaper from fine to micro-fine grain size, i.e., from 100 (140 µm) to 1500 (6 µm) mesh. Subsequently, it was polished with a 0.3 micron alumina suspension in a Labopol-5 polisher (Struers, Copenhagen, Denmark) with an 8 inch cloth at 400 revolutions per minute for 10 min. Finally, it was etched with an HNO_3_ solution (Nital 3) in C_2_H_6_O at 3% per volume for 5 s according to ASTM E-407 standard and observed with an Axio Observer inverted plate optical microscope (Carl Zeiss, Jena, Germany). From the images obtained, a microstructure quantitative analysis was performed, and the fractions of ferrite and pearlite present were determined with the free Image J software. Subsequently, microhardness tests were performed. Microhardness measurements were scanned with a Vickers MVK-HVL (Mitutoyo, Kawasaki, Japan) microdurometer with a 200 gf (1.96 N) and an application time of 15 s, the diamond indenter footprints were observed with a 40× objective lens. Measurements were made on the entire surface of each specimen with a distance between marks of approximately 0.25 mm.

### 3.2. Differential Dilatometry

To determine the austenite formation kinetics, dilatometry tests were performed with a vertical dilatometer L75 V (Linseis, Selb, Germany) with cylindrical specimens of 5-mm in diameter and 15-mm in length. The dilatometer uses an Electromechanical Transducer LVDT (Linear Variable Differential Transducer), with a resolution of 0.125 μm and an accuracy of 1% on the real scale. Each specimen contact surfaces were prepared by SiC sandpaper grinding, from extra to micro-fine (6–23 µm) and polished with 0.5 µm particle size alumina. Austenite formation was analyzed by pre-heating the specimens at a temperature of 35 °C with a heating rate of 0.08 °C s^−1^. Subsequently, the specimens were heated to a temperature of 1150 °C at different rate: 0.08, 0.33, 0.66 and 1.00 °C s^−1^, under an inert atmosphere of industrial argon at constant flow. Finally, the specimens were cooled at a rate of 0.42 °C s^−1^ to room temperature. The axial displacement of the specimen, time and temperature, were measured continuously during the tests, which were carried out in duplicate.

## 4. Results

### 4.1. Austenite Formation

**Figure 2:**
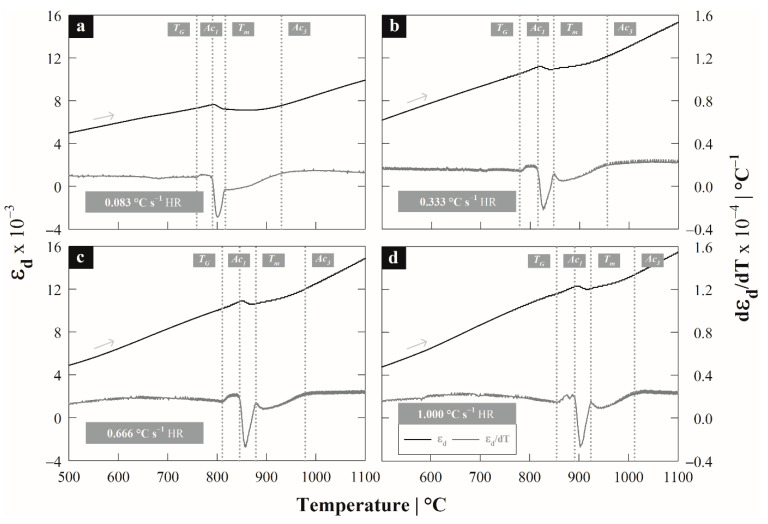
Dilatometric curves for 2.6Si Steel at different heating rates: (**a**) 0.08, (**b**) 0.33, (**c**) 0.66 y, (**d**) 1.00 °C s^−1^.

Austenite Figure 2a–d show the behavior of the dilation strain $\Delta L/L_{0}$ and its first derivative with respect to temperature $d\left(\Delta L/L_{0}\right)/dT$, where $L_{0}$ is the initial length of the specimen. The presence of three transformation zones is observed from the curve of the first derivative (dark gray line) delimited by the temperatures $Ac_{1}-T_{G}$, $T_{m}-Ac_{1}$ and $Ac_{3}-T_{m}$. The first zone is demarcated by $Ac_{1}-T_{G}$, where $T_{G}$ is associated with the beginning of a carbon precipitation stage as occurs in cast irons and silicon steels [1,14] and $Ac_{1}$ is the austenite formation start temperature. The second stage occurs between temperatures $T_{m}-Ac_{1}$; temperature $T_{m}$ is an intermediate temperature between the end of the pearlite decomposition stage and the beginning of the transformation of ferrite into austenite, similar to what occurs in low-carbon steels [1,2,4,22]. The third transformation stage starts at $T_{m}$ and extends until reaching the end temperature of austenite formation $Ac_{3}$; this stage corresponds to the transformation of ferrite into austenite, which is strongly influenced by the silicon content.

The mentioned stages are often present in steels with ferritic-pearlitic microstructures; austenite formation can occur from nucleation in pearlite colonies, with rapid growth that completely consumes the cementite followed by slow growth from the ferrite [22,23]. Moreover, the presence of two such marked stages associated with the formation of austenite differs from the behavior expected in unalloyed medium carbon steels where the formation of austenite occurs mainly in a single stage due to the overlapping during the transformation [5]. Nevertheless, the magnitude of the peaks associated with the transformation stages is different, since the first peak (first contraction, dark gray line) that appears is greater than the second. In association with the deconvolution model reported by Pawłowski [4] this behavior would imply that the transformation extension of the second stage is greater than the first, while the transformation rate is greater in the first than in the second due to the solubility of silicon in ferrite, cementite and austenite [24]. From the thermodynamic model proposed by Atkinson et al. [24] it is shown that silicon has low or no solubility in cementite in Fe-C-Si ternary systems and a high solubility in ferrite, especially at low supersaturation values, which causes the transformation into austenite to be delayed by the addition of silicon. As indicated, the effect of silicon falls mainly on the second stage where the transformation of ferrite into austenite occurs. Silicon is a strong stabilizer of the ferritic field, which causes a greater driving force to be required to carry out the transformation, which is obtained from the shift of critical temperatures to higher values, directly affecting the diffusivity of carbon and silicon on the ferrite.

On the other hand, when verifying the overheating required to start the austenite formation (first stage) as a function of the heating rate, the expression is considered $\Delta T^{S-I}=Ac_{1}\left(\varphi \right)-Ac_{1}^{0.08}$, while for the second $\Delta T^{S-II}=T_{m}\left(\varphi \right)-T_{m}^{0.08}$. In both cases, it is observed that the overheating by stages increases as a function of the heating rate, which indicates a greater amount of energy required. It should be noted that the criterion to establish $Ac_{1}^{0.08}$ y $T_{m}^{0.08}$ as the equilibrium temperatures within the ∆*T^S^*^−*i*^ terms is due to the low heating rate, mainly for the $Ac_{1}$ temperature; where *i* corresponds to stage I or II and indicates the transformation maximum rate.

This due to $Ac_{1}^{0.08}$ is approximately equal to the equilibrium value calculated with the semi-empirical equation of Andrews [25], which was compared with the $Ac_{1}^{0.08}≃Ae_{1}=$ 793.11 °C temperature. In Table 2, the critical temperatures compendium is indicated, as well as the overheating values by stages as a function of the heating rate.

### 4.2. Kissinger Method—Activation Energy

**Figure 3:**
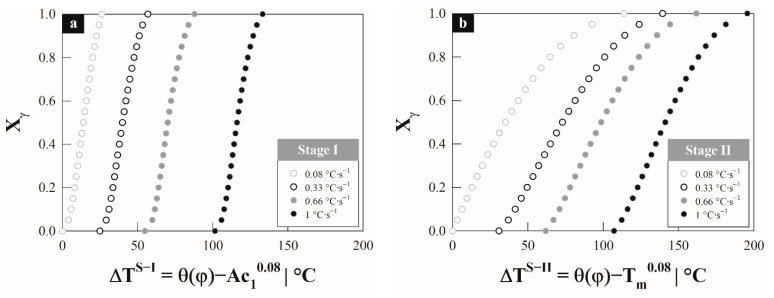
Austenite formation degree by stages as a function of overheating: (**a**) stage–I and (**b**) stage–II.

From the critical temperatures, the austenite formation degree $X_{\gamma }$ was determined with respect to the overheating by stages and as a function of the heating rate: $\Delta T^{S–I}=\theta (\varphi )-Ac_{1}{}^{0.08}$ and $\Delta T^{S–II}=\theta (\varphi )-T_{m}{}^{0.08}$ for the first and second stage, respectively. From these expressions, θ(φ) is the thermal path depend on the hetating rate corresponds to first and second stage interval as shown in Figure 3a,b. The austenite formation degree describes a sigmoid behavior and it was calculated employing the lever rule, linear regression method and first derivate criterion according to Vázquez–Gómez et al. [1]. In both stages the associated transformation requires a greater ∆*T^S^*^−*i*^ as the rate of heating increases (cf. Table 2). The difference in ∆*T^S^*^−*i*^ by stages is minimal depending on the heating rate; this could indicate that there is a similar driving force, which according to the type of transformation should be driven by the diffusion of carbon atoms in austenite under conditions local para- and ortho-equilibrium [26] due to the effect of silicon content. It should be noted that silicon slows the transformation during the first stage because it inhibits the dissolution of cementite, while in the second stage it hinders the diffusion of carbon into austenite.

To support the fact that austenite formation is promoted by the atomic diffusion of carbon and the effect of silicon on the diffusivity of carbon itself, the determination of the activation energy in stages is used. For this, it is necessary to calculate the austenite formation rate from the transformation degree using the following equation:

(8) $$\frac{dX_{\gamma }^{S-i}}{dt}=f{\left(X,T(t)\right)}_{\varphi }≃{\left(\frac{\Delta X_{\gamma }^{S-i}}{\Delta t}\right)}_{\varphi }$$

**Figure 4:**
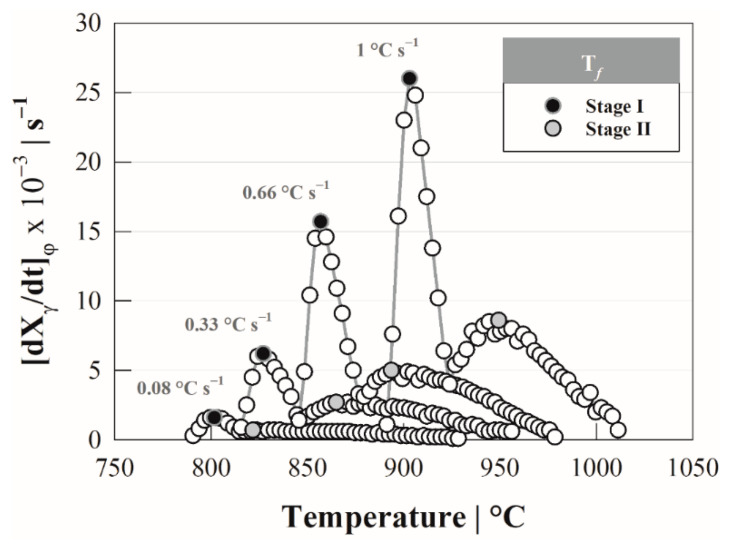
Austenite formation rate by stages as a function of heating rate: 0.08, 0.33, 0.66 and 1.00 °C s^−1^.

Figure 4 shows the behavior of the transformation rate showing the presence of two peaks associated with each of the austenite formation stages, whose maximum value is denoted by the temperature $T_{f}^{S-i}$. The amplitude and extent of the peak are associated with the transformation extension and heating rate; that is, the extent of transformation depends on the heating rate. During the first stage, the extension is defined as $\Delta T_{P\to \gamma }=T_{m}\left(\varphi \right)-Ac_{1}\left(\varphi \right)$, which increases as a result of the heating rate, while in the second, the extension is expressed by $\Delta T_{\alpha \to \gamma }=Ac_{3}\left(\varphi \right)-T_{m}\left(\varphi \right)$, which presents an opposite behavior (cf. Table 2).

From the temperatures of maximum transformation rate, the activation energies *Q* associated with the austenite formation by stages were obtained using the Kissinger method [27], which has been applied in solid–solid phase transformations under continuous heating conditions [14]. This method considers the temperature $T_{f}^{S-i}$ and the following expression:

(9) $$\ln\left[\frac{{\left(T_{f}^{S-i}\right)}^{2}}{\varphi }\right]=\frac{Q^{S-i}}{R\cdot T_{f}^{S-i}}+\ln\left(\frac{Q^{S-i}}{R\cdot k_{0}}\right)+\alpha$$

where *Q^S^*^−*i*^ is the activation energy for the stages *i* associated with the transformation mechanisms in kJ·mol^−1^, *k*_0_ is a pre-exponential factor and *α* is a constant related to the transformation degree.

**Figure 5:**
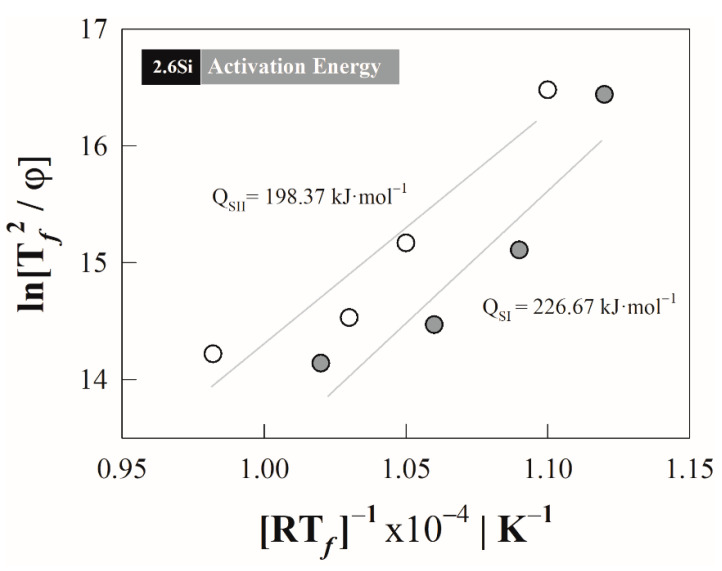
Activation energies in stages using the Kissinger method; the first and second stages are represented by grey and white circles, respectively.

The slope obtained from the mean of the set of values by plotting $\ln\left({\left(T_{f}^{S-i}\right)}^{2}/\varphi \right)$ vs. $1/R\cdot T_{f}^{S-i}$ as a function of the heating rate corresponds to the activation energy. In this case, the activation energies are: 226.67 and 198.37 kJ·mol^−1^ with a coefficient of determination (R^2^) of 0.8458 and 0.8971 for stage I and II, respectively. From Figure 5, it can be seen that the behavior is not completely linear due to the heating rate used in the method. In the case of the lowest rate of 0.08 °C s^−1^, the behavior tends towards an equilibrium condition, where the austenite formation is controlled mainly by the diffusion of substitutional elements [3], and therefore the behavior could differ from linearity.

### 4.3. Austenite Formation Non-Isothermal Model

**Figure 6:**
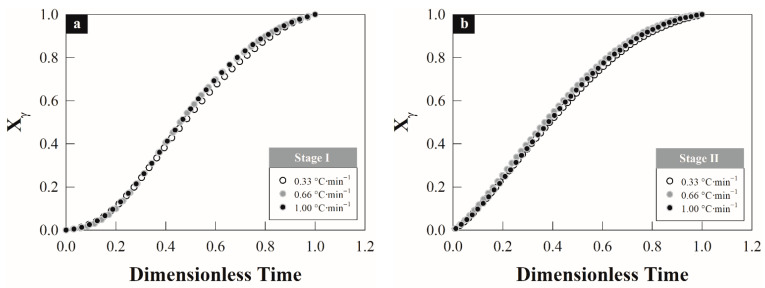
Behavior of the austenite formation degree by stages as a function of dimensionless time at different heating rates: 0.33, 0.66 and 1.00 °C s^−1^: (**a**) stage–I and (**b**) stage–II.

The main assumption regarding the application of the non-isothermal model is that the nucleation mechanism by stages does not change with the heating rate, since this mainly affects the parameter *k* associated with the transformation rate. Therefore, it is possible to obtain the behavior of the austenite formation degree independent of the heating rate, since during the transformation, time is the dependent variable. Figure 6 shows the behavior of the austenite formation degree by stages as a function of dimensionless time. In this case, the dimensionless time is calculated from the normalization of data associated with the transformation time:

(10) $$t_{d}=\frac{t_{T}^{i}}{t_{T}^{o}}$$

where $t_{d}$ is the dimensionless time, $t_{T}^{i}$ instantaneous transformation time and $t_{T}^{o}$ total transformation time. The assumption that the nucleation mechanism is invariant of the heating rate is fulfilled in Figure 6a,b, since the behavior of the austenite formation degree by stages maintains a constant trend (overlap condition).

The pre-exponential coefficient $k_{0}$ was calculated from the activation energy determined by the Kissinger method and Equation (6) under non-isothermal conditions as a function of heating rate. From this equation the value of $k_{0}$ was solved, obtaining the following expression:

(11) $$k_{0}^{S-i}=\frac{{\left(dX_{\gamma }^{S-i}/dt\right)}_{\varphi }}{n\left(1-X_{\gamma }^{S-i}\right){\left[\ln\left(\frac{1}{1-X_{\gamma }^{S-i}}\right)\right]}^{\frac{n-1}{n}}\left[\exp\left(-\frac{Q^{S-i}}{RT}\right)\right]}$$

The value of $k_{{}_{0}}^{S-i}$ was determined using the constant activation energy (cf. Figure 5) and the data associated with the transformation rate by stages ${\left(dX_{\gamma }^{S-i}/dt\right)}_{\varphi }$ (cf. Figure 4). Based on the assumption regarding the nucleation mechanism, a constant growth exponent n was proposed. The growth exponent is fixed by adjusting the $\ln k_{{}_{0}}^{S-i}$ until the behavior becomes independent of the heating rate [5]; i.e., when the same behavior of $\ln k_{0}$ is obtained as a function of the heating rate.

**Figure 7:**
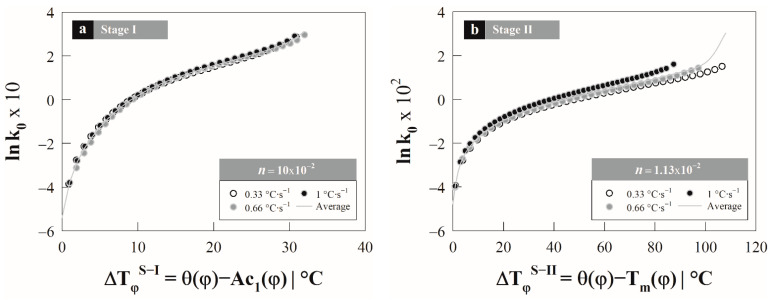
Behavior of the kinetic parameter $\ln k_{0}$ as a function of the heating rate by austenite formation stages: (**a**) stage–I and (**b**) stage–II.

Figure 7 shows the $\ln k_{{}_{0}}^{S-i}$ with respect to the instantaneous overheating as a function of the heating rate and for each stage of austenite formation: $\Delta T_{\varphi }^{S-I}=\theta \left(\varphi \right)-Ac_{1}\left(\varphi \right)$ and $\Delta T_{\varphi }^{S-II}=\theta \left(\varphi \right)-T_{m}\left(\varphi \right)$. The growth exponents $n^{S-i}$ that provide the best fit for $\ln k_{0}$ are: *n^S^*^−*I*^ = 10 × 10^−2^ y *n^S^*^−*II*^ = 1.13 × 10^−2^, for the first and second stage, respectively.

From the previous figure, it is observed that the adjustment agrees with the trend of the experimental data; however, there is a certain level of divergence in the behavior of the $\ln k_{0}^{S-i}$ in the second stage; due to this an average function of the $\ln k_{0}^{S-i}\to {\left(\ln k_{0}^{S-i}\right)}_{av}$ is established, considering the different heating rate values. Departing from function, the transformation degree is calculated using the temperature-dependent non-isothermal JMA model. In this case, the JMA model is expressed as:

(12) $$X_{\gamma }^{S-i}=1-\exp\left(-{\left(k_{0}^{S-i}\left(T\right)\right)}^{n^{S-i}}\cdot \exp\left(-\frac{n^{S-i}Q^{S-i}}{R\cdot T^{S-i}\left(\tau \right)}\right)\cdot \tau^{n^{S-i}}\right)$$

where the parameters $Q^{S-i}$ and $n^{S-i}$ are maintained constant for each stage, while the transformation temperature $T^{S-i}$ is calculated by means of the thermal path and therefore the heating rate,

(13) $$T^{S-i}\left(\tau \right)=\varphi \cdot \tau +T_{b}^{S-i}$$

where τ is the exclusive time of the transformation by stages and $T_{b}^{S-i}$ is the transformation start temperature as a function of the heating rate; $T_{b}^{S-I}\left(\varphi \right)=Ac_{1}\left(\varphi \right)$ for the first stage and $T_{b}^{S-II}\left(\varphi \right)=T_{m}\left(\varphi \right)$ for the second.

**Figure 8:**
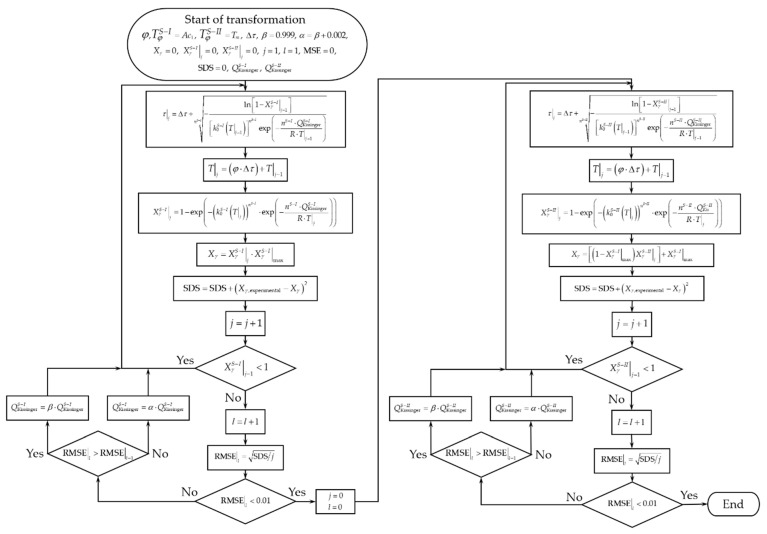
Solution algorithm using the non-isothermal model to estimate the austenite formation degree by stages.

Figure 8 shows the solution algorithm to apply the non-isothermal model. for this, the transformation degree was calculated using Equation (12). Initially, the critical transformation temperatures are established, $Ac_{1}$ and $T_{m}$ the step of time Δt and the heating rate *φ*, and the transformation degree is initialized by stages, ${X_{\gamma }^{S-I}|}_{j}$ and ${X_{\gamma }^{S-II}|}_{j}$, as well as the total transformation degree $X_{\gamma }$ in the step j=1. Subsequently, the condition is established to calculate the transformation degree in the first stage ${X_{\gamma }^{S-I}|}_{j}\geq 1$. Then the process starts with the transformation time ${\tau |}_{j}$, followed by the transformation temperature ${T^{S-I}|}_{j}$ and transformation degree ${X_{\gamma }^{S-I}|}_{j}$ using Equation (12) for the same step. The contribution of the first stage to the total transformation degree is established through the product ${X_{\gamma }^{S-I}|}_{j}\cdot {X_{\gamma }^{S-I}|}_{\max}$ using conservation law to determine ${X_{\gamma }^{S-II}|}_{\max}=1-{X_{\gamma }^{S-I}|}_{\max}=1-X_{pearlite}$. Finally, the total transformation degree is calculated as:

(14) $$X_{\gamma }={X_{\gamma }^{S-I}|}_{j}\cdot X_{P}+{X_{\gamma }^{S-II}|}_{j}\cdot \left(1-X_{P}\right)$$

**Figure 9:**
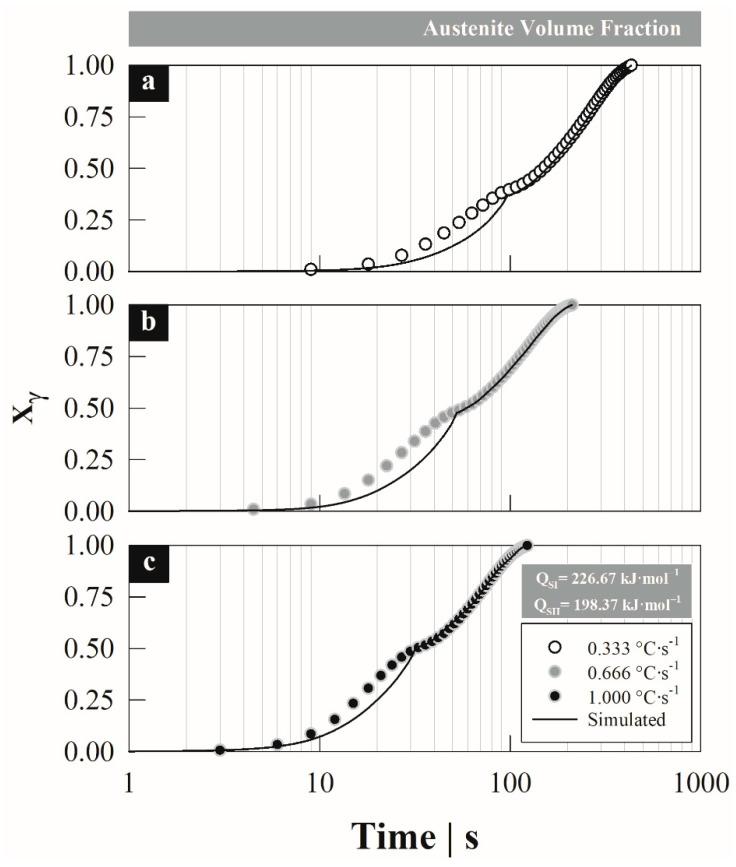
Comparison between the total austenite formation degree estimated with the non-isothermal model and experimental at different heating rates: (**a**) 0.33, (**b**) 0.66 y (**c**) 1.00 °C s^−1^.

Figure 9 compares the total austenite formation degree estimated with the non-isothermal model and the experimental data as a function of the heating rate, which describes a double sigmoid curve associated with the transformation by stages. Due to this, the behavior differs between one stage and another, mainly due to the formation mechanism, since each one of them occurs from a different constituent and nucleation site. This causes some divergence in the data estimated during the first stage when using the activation energy obtained by the Kissinger method. However, during the second stage, the fit of the estimated data agrees with the experimental data, overlapping both curves.

## 5. Discussion

As analyzed in the previous figure, the response of the non-isothermal model is not completely expected during the first stage, mainly due to activation energy obtained from the transformation rate calculated with the experimental data for each heating rate tested.

In this case, the activation energies were calculated with four heating rates in an interval of 0.08 to 1.00 °C s^−1^, and this causes a certain level of error when linearizing the peak temperature data (cf. Figure 5), since the term $1/R\cdot T_{f}^{S-i}$ with which the slope associated with the activation energy is obtained differs from a completely linear behavior. Therefore, the model only considers heating rates greater than 0.33 °C s^−1^, discarding the rate of 0.08 °C s^−1^. This heating rate is low enough that it can be approximated to an equilibrium and isothermal condition, so when considering it in the non-isothermal model, a divergence is generated in the estimated formation degree because the $\ln k_{0}$ function could not be adjusted to higher heating rates [5].

Conversely, the estimated response can be adjusted with the non-isothermal model through the iteration of the activation energy to minimize the root-mean-square error (RMSE) generated for the austenite formation degree estimated $X_{\gamma }^{est}$ and experimental $X_{\gamma }^{\exp}$ (cf. Figure 8) by means of,

(15) $$RMSE=\sqrt{\frac{\sum_{j=1}^{n}{\left({X_{\gamma }^{est}|}_{j}-{X_{\gamma }^{\exp}|}_{j}\right)}^{2}}{n}}<0.001$$

**Figure 10:**
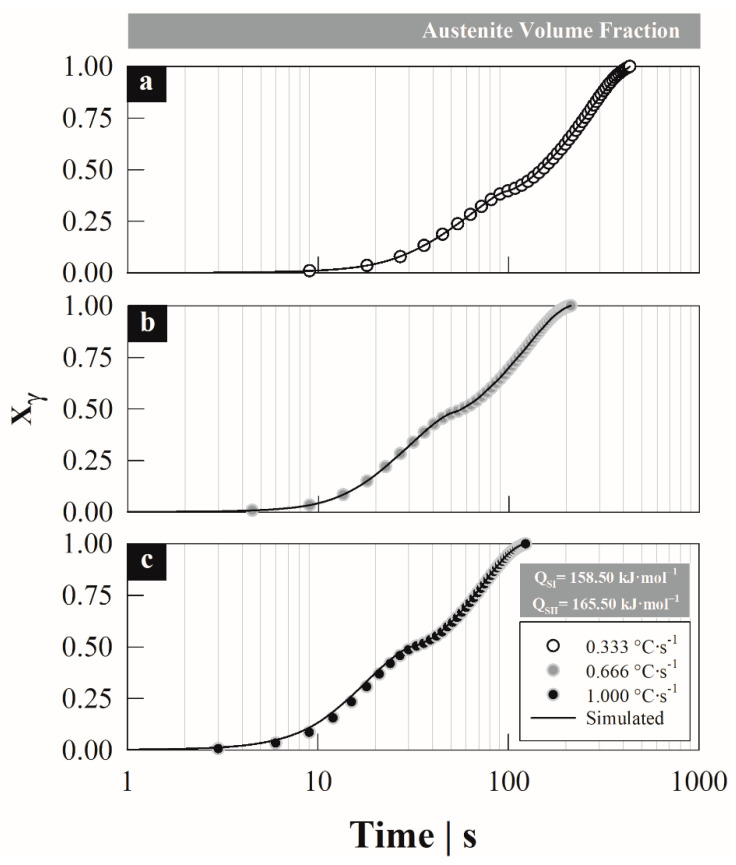
Correction of the total austenite formation degree estimated with the non-isothermal model, minimizing the root-mean-square error between the estimated and experimental data by activation energy in stages at different heating rates: (**a**) 0.33, (**b**) 0.66 and (**c**) 1.00 °C s^−1^.

Figure 10a–c shows the total austenite formation degree estimated with the non-isothermal model, minimizing the root-mean-square error as a function of the heating rate. It should be noted that the main advantage of applying the non-isothermal model is that the activation energy remains constant when changing the heating rate, which is considered an acceptable assumption in the sense that the mechanisms of austenite formation do not change by the heating rate.

The figures show an acceptable fit with a minimum error when comparing the estimated with experimental data, when using an activation energy of *Q^S^*^−*I*^ = 158.50 kJ·mol^−1^ and *Q^S^*^−*II*^ = 165.50 kJ·mol^−1^, for the first and second formation stage, respectively. These energies minimize the error between the estimated and experimental data, *RMSE*^0.33^ = 6.99 × 10^−3^, *RMSE*^0.66^ = 1.92 × 10^−3^ and *RMSE*^1.0^ = 9.12 × 10^−3^ (superscripts indicate heating rate). Despite having obtained these energies through an iteration technique, the values correspond to the energy associated with the driving force for the austenite formation; i.e., to the carbon diffusion in austenite-FCC with values between ~110–160 kJ·mol^−1^ for silicon-alloyed steels [14].

## 6. Conclusions

A non-isothermal transformation model was implemented to determine the austenite formation kinetics by stages in a silicon-alloyed steel (2.6% wt. Si). The degree and the austenite formation rate were determined by dilatometric analysis. It was observed that the austenite formation rate presents two peaks, which are associated with each of the austenite formation stages, and that correspond to the maximum transformation rate dependent on the heating rate. With these data and the Kissinger method, the activation energies associated with austenite formation were obtained by stages, from the temperatures of maximum austenite formation rate. The austenite formation degree by stages was estimated with the non-isothermal transformation model, observing a good approximation for the second stage and with some deviation in the first. In turn, the estimated degree of austenite formation was adjusted with the experimental one, optimizing the activation energy by minimizing the root-mean-square error. Activation energies of 158.50 and 165.50 kJ·mol^−1^ were obtained from the optimization for the first and second stages, respectively, which correspond to the activation energy associated with the carbon diffusion in austenite in silicon-alloyed steels.

## Figures and Tables

**Table 1:** Chemical composition of silicon-alloyed steel.

Steel	C	Si	Mn	P	S
2.6Si	0.27	2.60	0.59	0.02	0.03

Values are shown in % wt.

**Table 2:** Critical austenite formation temperatures as a function of heating rate: 0.08, 0.33, 0.66 and 1.00 °C s^−1^. ∆*T^S–I^*, overheating–first stage; ∆*T^S–II^*, overheating–second stage; ∆*T_P_*_+*α*→*γ*_, overall transform extension.

HR	*T_G_*	$Ac_{1}$	*T_m_*	$Ac_{3}$	∆*T^S–I^*	∆*T^S–II^*	∆*T_P_*_+*α*→*γ*_
0.08	757.6	790.7	816.7	931.4	0.0	0.0	140.7
0.33	781.6	816.6	847.5	956.1	25.9	30.8	139.5
0.66	813.4	845.6	878.5	978.5	54.9	61.8	132.9
1.00	855.9	891.3	923.9	1012.3	100.6	107.2	121.0

Values of heating rate and temperatures are shown in °C s^−1^ and °C, respectively.

## Data Availability

The data presented in this study are available on request from the corresponding author.
